# Use of Stay-at-Home Orders and Mask Mandates to Control COVID-19 Transmission — Blackfeet Tribal Reservation, Montana, June–December 2020

**DOI:** 10.15585/mmwr.mm7014a3

**Published:** 2021-04-09

**Authors:** Caroline Q. Pratt, Anna N. Chard, Rosaula LaPine, K. Webb Galbreath, Cinnamon Crawford, Albert Plant, Garland Stiffarm, Neil Sun Rhodes, Lorissa Hannon, Thu-Ha Dinh

**Affiliations:** ^1^Epidemic Intelligence Service, CDC; ^2^CDC COVID-19 Emergency Response Team; ^3^Blackfeet Nation, Browning, Montana; ^4^Indian Health Service, Browning, Montana.

COVID-19 has disproportionately affected persons who identify as non-Hispanic American Indian or Alaska Native (AI/AN) (*1*). The Blackfeet Tribal Reservation, the northern Montana home of the sovereign Blackfeet Nation, with an estimated population of 10,629 (*2*), detected the first COVID-19 case in the community on June 16, 2020. Following CDC guidance,* and with free testing widely available, the Indian Health Service and Blackfeet Tribal Health Department began investigating all confirmed cases and their contacts on June 25. The relationship between three community mitigation resolutions passed and enforced by the Blackfeet Tribal Business Council and changes in the daily COVID-19 incidence and in the distributions of new cases was assessed. After the September 28 issuance of a strictly enforced stay-at-home order and adoption of a mask use resolution, COVID-19 incidence in the Blackfeet Tribal Reservation decreased by a factor of 33 from its peak of 6.40 cases per 1,000 residents per day on October 5 to 0.19 on November 7. Other mitigation measures the Blackfeet Tribal Reservation used included closing the east gate of Glacier National Park for the summer tourism season, instituting remote learning for public school students throughout the fall semester, and providing a Thanksgiving meal to every household to reduce trips to grocery stores. CDC has recommended use of routine public health interventions for infectious diseases, including case investigation with prompt isolation, contact tracing, and immediate quarantine after exposure to prevent and control transmission of SARS-CoV-2, the virus that causes COVID-19 (*3*). Stay-at-home orders, physical distancing, and mask wearing indoors, outdoors when physical distancing is not possible, or when in close contact with infected or exposed persons are also recommended as nonpharmaceutical community mitigation measures (*3*,*4*). Implementation and strict enforcement of stay-at-home orders and a mask use mandate likely helped reduce the spread of COVID-19 in the Blackfeet Tribal Reservation.

The potential effects of community mitigation measures on changes in the number and incidence of new COVID-19 cases in the Blackfeet Tribal Reservation during June 16–December 10, 2020, were assessed using deidentified laboratory and case investigation data. The tribal health clinic, the Indian Health Service, a dialysis clinic, and a long-term care facility performed testing for SARS-CoV-2 and used various data collection tools. Local public health nurses abstracted case investigation data, including patient age, sex, race, ethnicity, test date, and exposure information. A case was defined as receipt of a positive SARS-CoV-2 result from either a nucleic acid amplification test, such as a polymerase chain reaction test, or a rapid antigen detection test by a resident of the Blackfeet Tribal Reservation. Incidence was calculated as the daily number of new COVID-19 cases per 1,000 residents. Analyses were conducted using SAS (version 9.4; SAS Institute). Population estimates for the Blackfeet Tribal Reservation and for Montana were obtained from the U.S. Census Bureau (*2*,*5*). This activity was reviewed by CDC and was conducted consistent with applicable federal law and CDC policy.^†^

During 2020, the Blackfeet Nation implemented three stay-at-home orders; mask use in public was required by all three orders. The first was a mandatory stay-at-home order,^§^ which was in place during June 29–July 31; violations of isolation or quarantine orders could result in a fine up to $500.^¶^^,^** The second was a recommended stay-at-home order,^††^ which began August 19. The third was an enforced stay-at-home order,^§§^ which began September 28. Under this third order, breaking quarantine or isolation orders could result in up to 3 years in jail and a fine up to $5,000.^¶¶^ Patients unable to isolate at home were provided temporary housing in two local hotels. A COVID-19 dispatch team delivered medications and food to community members, as needed.

During June 16–December 10, 2020, a total of 1,180 COVID-19 cases were reported in the Blackfeet Tribal Reservation (Table). The median age of patients was 36 years (range = 0–96 years); 50.5% of cases occurred in females, and 91.9% of patients self-identified as AI/AN. After the first COVID-19 case was reported in the community on June 16, the Blackfeet Tribal Business Council voted not to open the east gate of Glacier National Park, which borders the reservation, through the end of the 2020 tourist season (*6*). The Blackfeet Tribal Reservation recorded few cases during July, when mandatory stay-at-home orders and ongoing case investigation and contact tracing were in effect, with an average daily incidence of 0.10 cases per 1,000 residents (Figure 1). On July 31, the Blackfeet Tribal Reservation opened its campgrounds to residents when the mandatory stay-at-home orders expired. In August, a slight increase in incidence was observed, to 0.19 cases per 1,000.

**TABLE:** Characteristics of Blackfeet Tribal Reservation residents and COVID-19 patients — Blackfeet Tribal Reservation, Montana, June–December 2020

Characteristics	No. (%)	
	All residents* (N = 10,629)	COVID-19 patients† (N = 1,180)
**Age, yrs**		
Mean (SD)	N/A	37.8 (20.7)
Range	N/A	0–96
Median (IQR)	30.4	36 (21–54)
**Sex**		
Female	5,257 (49.5)	596 (50.5)
Male	5,372 (50.5)	564 (47.8)
Unknown	0 (—)	20 (1.7)
**Race^§^**		
American Indian or Alaska Native	8,865 (83.4)	772 (91.9)
Asian	8 (0.1)	1 (0.1)
Black or African American	24 (0.2)	1 (0.1)
Multiple races	112 (1.1)	20 (2.4)
Other race	125 (1.2)	23 (2.7)
Unknown	0 (—)	18 (2.2)
White	1,482 (13.9)	5 (0.6)
**Ethnicity^¶^**		
Hispanic	206 (1.9)	1 (0.1)
Non-Hispanic	10,423 (98.1)	548 (74.2)
Unknown	0 (—)	190 (25.8)

**Abbreviations:** IQR = interquartile range; N/A = not available; SD = standard deviation.

* https://www.census.gov/tribal/?aianihh=0305

† Blackfeet case investigation report.

^§^ Unknown for 340 COVID-19 patients.

^¶^ Unknown for 441 COVID-19 patients.

**FIGURE 1:**
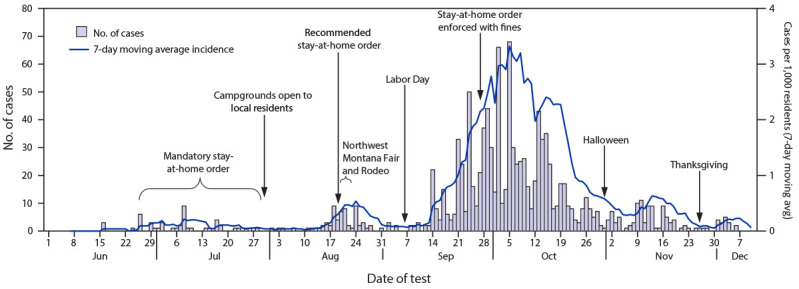
Number of COVID-19 cases, by test date and 7-day moving average incidence (N = 1,150) — Blackfeet Tribal Reservation, Montana, June 1–December 10, 2020*^,†^ * Case data collected and recorded by Blackfeet and Indian Health Service public health nurses. ^†^ Among 1,180 total cases, 30 were missing test date and are not included in the figure.

The second, or recommended, stay-at-home order commenced on August 19. However, the number of cases increased after gatherings at the Northwest Montana Fair and Rodeo (August 19–23) in Kalispell, outside of the reservation, and during Labor Day weekend (September 5–7). Daily incidence peaked at 6.40 cases per 1,000 residents on October 5, which was 63 times the incidence in July.

On September 28, a third stay-at-home order was issued, with strict enforcement and substantial fines for violation. Afterward, incidence decreased to 0.19 cases per 1,000 by November 7. A gradual increase in newly identified cases among persons aged 5–17 years and 30–39 years began the week of August 9, after the campgrounds opened on July 31, and peaked the week of August 16 (Figure 2). During August, the numbers of cases in these age groups were higher than those in other age groups. Incident cases among persons aged 18–39 years and 50–64 years increased after the Northwest Montana Fair and Rodeo (week of August 16) and Labor Day weekend (week of September 6), and peaked during the week of September 27, before the enforced stay-at-home order was issued.

**FIGURE 2:**
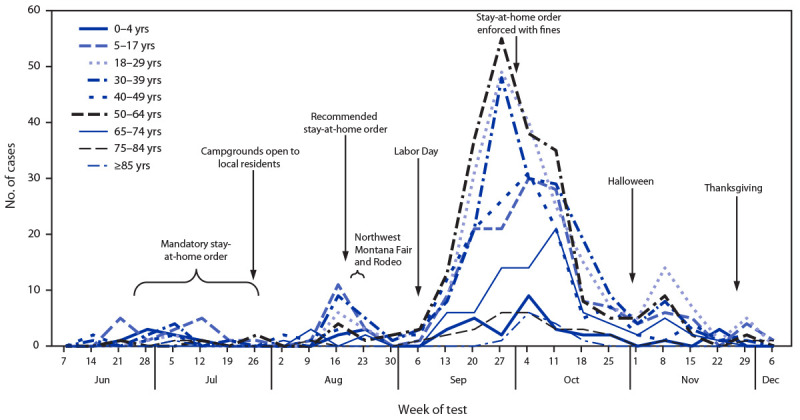
Number of weekly COVID-19 cases, by week of test and age group (N = 1,150) — Blackfeet Tribal Reservation, Montana, June 1–December 10, 2020*^,†^ * Case data collected and recorded by Blackfeet and Indian Health Service public health nurses. ^†^ Among 1,180 total cases, 30 were missing test date and are not included in the figure.

Among 142 (12.0%) of 1,180 patients with available household exposure data, 121 (85.2%) reported at least one household contact with COVID-19. Workplace exposure data were available for 198 (16.8%) patients, 12 (6.1%) of whom reported a workplace exposure. Community exposure data were available for 133 (11.3%) patients; among these, 53 (39.8%) reported known community exposure. Twelve patients (1.0%) reported exposure in an adult congregate living facility.

## Discussion

After implementation of mitigation measures, including case investigation, contact tracing, a mandatory stay-at-home order, and required mask use in public, the average reported daily COVID-19 incidence in the Blackfeet Tribal Reservation remained low (0.10 cases per 1,000 residents) during July. When the mandatory stay-at-home order expired on July 31, the Tribal Business Council issued a recommended stay-at-home order on August 19. After the opening of local campgrounds and Northwest Montana Fair and Rodeo and Labor Day weekend gatherings, daily COVID-19 incidence increased sharply, peaking October 5, and representing a sixty-three-fold increase over the daily average incidence in July. The continued increase in newly identified cases after September 28, when the enforced stay-at-home order commenced, reflects exposures that occurred in the preceding 2 weeks.*** The strictly enforced stay-at-home order, with increased penalties, likely contributed to the more than thirtyfold decrease in incidence by November 7.

The steep declines in COVID-19 incidence in the Blackfeet Tribal Reservation might not have occurred without widespread and consistent enforcement of the mandate for mask use in public and stay-at-home orders. Wearing a mask reduces SARS-CoV-2 transmission from persons with symptomatic or asymptomatic infection and offers some protection for the wearer.^†††^ On July 15, Montana first implemented a limited mask use mandate, which only applied to counties with four or more active COVID-19 cases,^§§§^ but enforcement across the state was inconsistent (*7*). COVID-19 incidence in Montana increased throughout September and October, peaking November 14, at 1.54 cases per 1,000 residents (*8*). After the mask use mandate was applied to all Montana counties on November 17 (*9*), incidence in the state decreased (*8*).

The increases in COVID-19 cases among Blackfeet residents aged 5–17 years and 30–39 years followed relaxation of stay-at-home orders, the opening of campgrounds, and gatherings at the Northwest Montana Fair and Rodeo and during Labor Day weekend. The peaks in COVID-19 incidence in these groups were followed approximately 6 weeks later by a peak among persons aged 50–64 years. The average household size in the Blackfeet Tribal Reservation (3.4 persons) is higher than that in Montana (2.4 persons) (*2*,*5*). Using limited available household data (available for 12% of all cases), a household COVID-19 contact was reported for a larger proportion of cases among the Blackfeet (85%) than for cases among other Montana residents (22%) (*10*). Multigenerational households might contribute to COVID-19 transmission between age groups in the Blackfeet Tribal Reservation; however, information on multigenerational households for the Blackfeet was not available. Future planning for mitigation measures and data collection should take multigenerational households into account.

The findings in this report are subject to at least five limitations. First, the different performance characteristics of the two diagnostic tests (rapid antigen detection and molecular SARS-CoV-2 tests) created potential misclassification of cases. Second, complete standardized data were not available because the various entities conducting testing did not use the same data collection tools. Third, the lack of consistently collected data on contact tracing, exposures, order compliance, and relationships between COVID-19 cases prevented the assessment of secondary transmission. Fourth, data on household, workplace, and community exposure was limited; comparison with other populations should be made with caution. Finally, the relative contribution of each mitigation measure to the changes in COVID-19 rates could not be ascertained.

The enforcement of stay-at-home orders, coupled with a mandate for mask use in public, likely contributed to a reduction in COVID-19 incidence, potentially helping to control the pandemic in the Blackfeet Tribal Reservation. A combination of mitigation measures, including case investigation, contact tracing, and enforced stay-at-home and mask use orders, will likely reduce COVID-19 transmission by limiting potential exposure to SARS-CoV-2. As of 2021, vaccination is available and recommended as another effective method of COVID-19 mitigation. In communities disproportionately affected by COVID-19, these mitigation strategies are likely to help reduce some COVID-19–associated health disparities.

**Summary**

**What is already known about this topic?**

Community mitigation measures (e.g., stay-at-home orders and mask use), coupled with case investigation and contact tracing with immediate isolation or quarantine, are primary approaches to preventing and controlling community SARS-CoV-2 transmission.

**What is added by this report?**

In the Blackfeet Tribal Reservation, enforcement of stay-at-home orders and mandated use of face coverings in public, with potential fines and jail for noncompliance, were associated with a thirty-three-fold reduction in COVID-19 incidence from its peak of 6.40 cases per 1,000 residents per day on October 5 to 0.19 on November 7, 2020.

**What are the implications for public health practice?**

Enforcement of stay-at-home orders and mask use mandates, coupled with robust public health investigations, have been shown to reduce COVID-19 incidence.

## Footnotes

*
https://www.cdc.gov/coronavirus/2019-ncov/php/contact-tracing/contact-tracing-plan/overview.html

† 45 C.F.R. part 46, 21 C.F.R. part 56; 42 U.S.C. Sect. 241(d); 5 U.S.C. Sect. 552a; 44 U.S.C. Sect. 3501 et seq.

§
http://www.blackfeetnation.com/wp-content/uploads/2020/07/22.-Blackfeet-Tribe_Resolution_Re-affirming-Closure-of-Blackfeet-Tribe-in-Response-to-COVID-19-Outreach_June-29_2020.pdf

¶
http://www.blackfeetnation.com/wp-content/uploads/2020/07/24.-Blackfeet_Resolution-282-200_Approving-Amende-Quarantine-Order.pdf

**
http://www.blackfeetnation.com/wp-content/uploads/2020/07/27.-Blackfeet_Resolution-285-2020_Enacting-Isolation-Order.pdf

††
http://www.blackfeetnation.com/wp-content/uploads/2020/08/20.-Blackfeet-Resolution_Extending-closure-until-further-notice.pdf#:~:text

§§
http://blackfeetnation.com/covid19/

¶¶
http://www.blackfeetnation.com/wp-content/uploads/2020/10/Blackfeet-Tribe-Resolution_Continuing-Current-Fines-and-Offenses-beginning-on-October-26-2020-until-Further-Notice-Under-Current-Phase-Restrictions.pdf

***
https://www.cdc.gov/coronavirus/2019-ncov/hcp/clinical-guidance-management-patients.html

†††
https://www.cdc.gov/coronavirus/2019-ncov/prevent-getting-sick/cloth-face-cover-guidance.html

§§§
https://dphhs.mt.gov/aboutus/news/2020/directiverequiringfacecoverings
